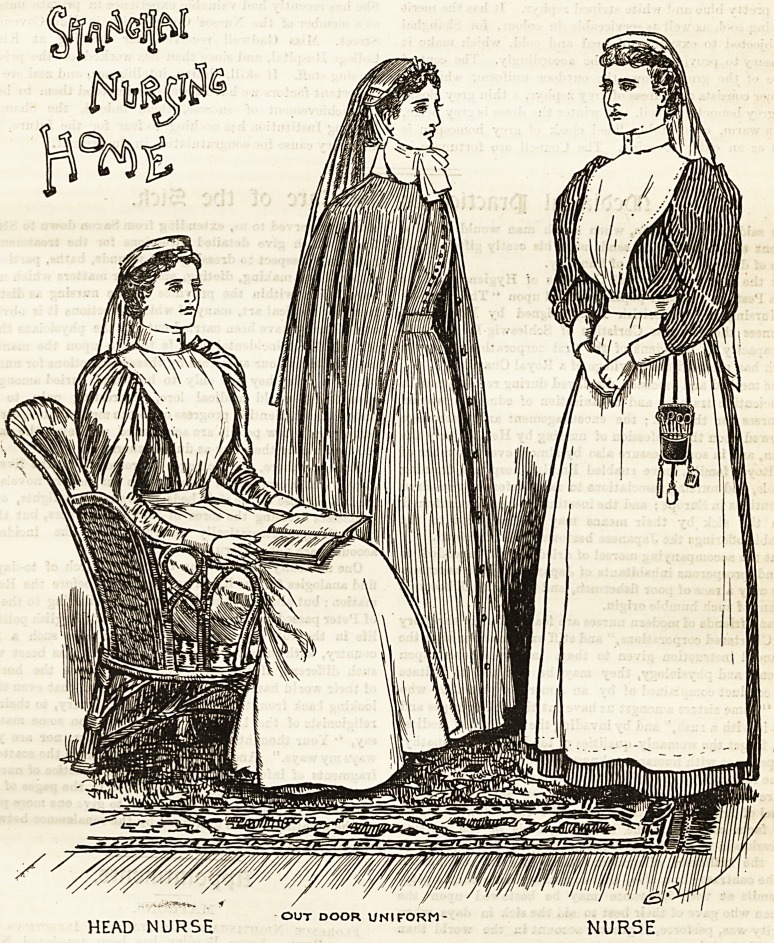# The Hospital Nursing Supplement

**Published:** 1896-10-03

**Authors:** 


*The Hospital*, Oct 3, 1896. *Extra Supplement.*

### "The Hospital" Nursing Mirror.

Being the Extra Nursing Supplement of "The Hospital" Newspaper.

[Contributions for this Supplement should be addressed to the Editor, The Hospital, 28 & 29, Southampton Street, Strand, London, W.C., and should have the word "Nursing" plainly written in left-hand top corner of the envelope.]

### News from the Nursing World.

### SIR JOSEPH LISTER AND THE LIVERPOOL NURSES.

While Sir Joseph Lister was in Liverpool the other day he visited the Royal Southern Hospital, and, escorted by Dr. Rawdon, went round the wards and also inspected the nurses' quarters and the sanitary arrangements. Afterwards there was a gathering in the theatre, and the following address on behalf of the nursing staff was read: "Sir Joseph Lister, we welcome you to the Royal Southern Hospital, not only for what you have done, by your careful researches, towards preserving the lives and mitigating the sufferings of patients all over the world, but we, a small representative body of nurses, tender to you our deep gratitude for one result of your work of great importance to us—viz., that many of our number who were formerly liable to much inconvenience, and often, alas! to contract fatal illnesses through attending the wounded, now find that, owing to your researches, the risks are reduced to a minimum. Again tendering to you our heartfelt thanks, and wishing you a long life, we are, &c." Before leaving, Sir Joseph Lister made this entry in the visitors' book: "Joseph Lister has been much gratified by seeing this fine hospital so well appointed in all respects, as well as by the very kind reception he has met with from the governing body and his brethren on the staff. September 22nd, 1896."

### THE NEW NURSES' HOME AT CHELSEA INFIRMARY.

The new Home, which will be ready to accommodate the nursing staff of the Chelsea Infirmary in a few weeks' time, is a comfortable and well-contrived building, in which every nurse and probationer will have a charming little room to herself, while there is liberal space provided for recreation and class-rooms. The night nurses will inhabit the top floor, shut off by doors so that no sound from below can disturb their rest. Miss de Pledge has thought out a very cosy arrangement by which on the floor immediately below is a sitting-room for the night nurses' use, in which they can have their meals served from a special little kitchen, and come down to them without being obliged to put on uniform—a great comfort and saving of precious off-duty time. The bed-room floors are stained and polished, while the corridors are laid with wood blocks, unpolished and noiseless. The furnishing, except for very spacious hanging cupboards built into every room, still remains to be completed.

### GUY'S HOSPITAL LADIES' ASSOCIATION.

This useful society is just a year old, and the first annual meeting will be held in the coming week, when a report of the work accomplished will be presented. The association was started with the threefold object of providing clothes for destitute patients when discharged from the hospital; of regular visiting in the wards by members willing to help in this way, the visiting to be of a friendly and social character; and of taking up any work in connection with the hospital which may commend itself to a general meeting. Membership is honorary or working, the subscription being half a guinea annually in the first case and half-a-crown in the second. In connection with the clothes distribution working parties are held during the winter months at the houses of the members, and garments made are left in the hands of Miss Nott-Bower and the sisters. Mrs. Lauriston Shaw has acted as secretary during the past year, and to her, at 10, St. Thomas's Street, S.E., should be sent the names of ladies desirous of joining the association and willing to visit in the wards. Gifts of old clothes and flowers and fruit for the patients will be gladly welcomed at any time.

### INTERNATIONAL WOMEN'S CONGRESS.

In any discussions relating to women's work the subject of nursing as an occupation naturally finds a place. At the International Women's Congress, now being held in Berlin, a paper on "Sick Nursing as a Vocation for Women," was read by Head Matron Stosch, of Berlin. An account of the nursing work carried on in the Colonies by the German Women's Union was contributed by Miss Mueseler, also a resident in Berlin.

### ST. ANDREW'S CONVALESCENT HOSPITAL, CLEWER.

A bazaar is being organised, to take place at this hospital on November 26th, towards which contributions will be very gladly received by the Sister Superior. All kinds of gifts will be welcome, especially clothing for the poor, knitted garments, and fancy articles, or materials for making up into garments, cakes for a cake stall, or any farm and dairy produce. Residents in Windsor and the neighbourhood, who know something both of this hospital and of the many good works which owe their origin to the Community of St. John the Baptist, should respond with prompt promises of help. We hope contributions of all kinds may flow in during the next few weeks, and that the bazaar will result in some substantial help for the hospital.

### THOUGHTFUL REMEMBRANCE.

The District Nursing Association of St. Agnes, Cornwall, has just received a gift of £8 5s., collected amongst a number of "St. Agnes boys" now far away in South Africa, and forwarded, with a list of subscribers, by Mr. N. Wenmoth to Mrs. Littleton Geach, a member of the association committee. It is a pleasant proof of kindly recollection which will be deservedly valued.

### BOLTON DISTRICT NURSING ASSOCIATION.

The work of this association has steadily increased year by year since its first beginnings in 1889. The number of cases attended that first year was 303, whilst for 1895 they stand at 1,283. Owing to the large demand for the nurses' services, an extra nurse had to be engaged during the second half of last year, and the committee have come to the conclusion that this eighth nurse ought to be established permanently upon the staff. It is unfortunate that subscriptions should have decreased by

£30 in 1895, but the record of work in the annual report will surely lead to a speedy correction of this falling off and additional help in the present year. The nurses had a heavy time last autumn in dealing with an outbreak of typhoid fever, and two of them contracted the disease, but have happily recovered and returned to their work.

### MADAME PATTI'S ANNUAL CONCERT.

Madame Adelina Patti has given her annual charity concert at Cardiff this year, instead of at Swansea, as heretofore. The entertainment took place in the Park Hall, and the proceeds, which realised £800, will be devoted to the benefit of "the Cardiff Infirmary and the poor in the neighbourhood of Craig-y-Nos."

### A NEW COTTAGE HOSPITAL.

Through the kindness of Mrs. Parr, of Killichronan, a small cottage hospital has been started near Oban, at a place called Gleneritten. Mrs. Parr has herself defrayed the entire cost of the building and furnishing, and has further promised to give £100 annually towards the expenses of the nursing staff, so that it is to be expected that the West Highland Cottage Hospital, as it is called, will be adequately staffed, which is not always the case at these small institutions, where the nursing is not infrequently in the hands of one nurse, who is expected to perform day and night duty.

### ODDFELLOWS AND THE LICHFIELD NURSING INSTITUTION

At a recent parade of the Lichfield Oddfellows at the Cathedral, the alms collected after the service were devoted to the Lichfield Nursing Institution. The Bishop of Lichfield preached the sermon, and in the course of it remarked that he "could commend the institution to the charitable feelings of those present. It provided a nurse who was able to attend upon the sick poor throughout the city, and who was constantly labouring amongst them." He asked for liberal gifts towards the maintenance of an institution that gave the poor the very best care that could be provided in their times of sickness.

### THE TROUBLES AT TOTNES.

It will be remembered that the Workhouse Infirmary Nursing Association were recently compelled to withdraw their nurses from this union infirmary, two of them declining to remain in consequence of the treatment they received. These nurses worked at the infirmary for a month, however, but the guardians have declined to pay them for that time or to refund their travelling expenses, and are persisting in this refusal in spite of the representations of the association. They also have refused a testimonial to the late head nurse, though there was no complaint against her respecting the manner in which she discharged her duties. The Totnes Guardians have now cheerfully handed over the unfortunate inmates of the infirmary wards to the ministrations of untrained women.

### NURSING IN AUSTRALIA.

The Australian Government, having decided to withdraw their subsidies from hospitals which do not adopt an eight hours' working day for their nursing staffs, the authorities of charitable institutions in Australia are naturally in a state of much dismay. A deputation, representing various hospitals in Melbourne, waited the other day upon the Premier to utter a protest against this drastic measure, and received for reply a promise that the new regulation should not be put into force for six months. In the meantime, the Premier intimated that he would be glad to hear from themselves the views of nurses on the subject, an invitation in replying to which members of the nursing profession in Australia should lose no time. It will be interesting to know how nurses in Australia regard the proposed alterations, and they certainly have a unique opportunity offered to them of expressing their real feelings in the matter.

### FANCY FAIR AT THE GUEST HOSPITAL.

Extensive alterations and improvements are being carried on at the Guest Hospital, Dudley, at an estimated cost of about £3,000, and to help towards the cost of furnishing, a garden party and fancy fair took place on September 16th. There were bicycle races and maypole dances, and, in spite of bad weather, the entertainments were well patronised, and some £50 profit has resulted for the hospital.

### LADY GUARDIANS FOR IRELAND.

The first lady to obtain a seat on an Irish Board of Guardians is Miss Martin, of Enniskillen, and her election would appear to be popular, as it is said that her first appearance in the board-room was greeted by her fellow-Guardians with cheers. The Bill to enable women to act in this capacity in Ireland was introduced by Mr. W. Johnston, of Ballykilbeg. The great work of reform in Irish workhouses may be greatly helped and hastened by the presence of sensible women on the boards of guardians.

### SHORT ITEMS.

Viscountess Boyne opened the new Bridgnorth and South Shropshire Infirmary on the 16th inst.—Nurse Lois Lightowler, who is leaving the Toxteth Infirmary, Liverpool, where she has worked for some time as charge-nurse and night superintendent, was presented the other day by the night nurses and probationers with a handsome writing case with gold monogram, as a "mark of regard and esteem."—Miss Beatrice Garvie, L.R.C.P., L.R.C.S.Edin., L.F.P.S.Glasg., has been appointed clinical assistant at the New Samaritan Hospital, Glasgow.—Miss A. Elliott has been appointed to the post of sanitary inspector for the Vestry of St. George's, Southwark. Miss Elliott comes from St. Helens, Lancashire.—Dr. Lucinda Key, the second woman to graduate at the Tennessee Medical College, has recently died at Chattanooga, U.S.A., where she carried on a successful practice.—Sister Josephine, who has for nineteen years superintended the nursing at the General Infirmary, Doncaster, is about to retire from her post, and the people of Doncaster are taking steps to present her with a testimonial. The quarterly meeting of the general committee of the Aberdeen District Nursing Association, in affiliation with the Q.V.J.I., was held on September 14th. Miss Peter, Inspector of Queen's Nurses, was present.—On October 15th a "Cyclists' Carnival" is to be held at Taunton, the proceeds of which are to be divided between the Taunton and Somerset Hospital and the District Nursing Association.—A very successful concert in aid of the Kingussie, Alvie, and Insh Nursing Association was held in Kingussie, Inverness, a week or two since. Mrs. Brewster Macpherson, of Balavil, is president of the association.—We have to acknowledge, with thanks, the receipt of 2s. 6d. from Nurse Dunwodie as her subscription to The Hospital Convalescent Fund.

### Hygiene: For Nurses.

By John Glaister, M.D., F.F.P.S.G., D.P.H.Camb., Professor of Forensic Medicine and Public Health, St. Mungo's College, Glasgow, &c.

### XXVI.—ATMOSPHERE OF HOUSE-DRAIN AND SEWER.—MODES OF TESTING DRAINS.—SEWER VENTILATION.

Frequent mention of the foul air of soil pipe, house-drain, and sewer, and of its being a disease-producing factor, has already been made. Let us briefly inquire wherein the air of these parts of the sanitary apparatus is harmful. In the first place, it is to be remembered that these channels carry off materials in a putrescible condition from which, when putrescence takes place, as it readily does in water, foul smelling gases are disengaged, which are chiefly composed of compounds of sulphur with hydrogen, or ammonia, or both, as in a rotten egg. From constant exposure to these alone ill-health is more or less quickly engendered, shown by loss of appetite, malaise, weakness, pallor of countenance, and not infrequently, eruptive skin diseases. When present in concentrated form, as in sewers, they produce alarming symptoms, and sometimes fatal results. In addition to these malodorous gases there are other equally objectionable gases generated, which, however, only possess a faint, sickly, or fœtid odour. These are compounds of carbon and ammonia, and carbon and hydrogen. Of greater importance still than any of these are certain disease-producing micro-organisms. Diverse opinions are held in respect of the amount of mischief produced by the agency of sewer air; but little doubt need be entertained on the question, since, indubitably, sewer-air is harmful. There is no question, however, regarding the potency for harm of certain micro-organisms which are found associated with decomposing fæcal matter, and particularly with the discharge from patients suffering from enteric fever and diphtheria. Entrance of gases from soil-pipe or house-drain, and more certainly of these micro-organisms, into the atmosphere of occupied rooms, will sooner or later set up ill-health or disease, and it is to prevent these entering that water-traps are used, since water arrests and holds in solution and suspension such gases and micro-organisms. A dry pipe or trap is, therefore, more dangerous than a wet one, and the atmosphere of sewer, drain, or soil-pipe is always more dangerous in droughty weather than in wet, and in the case of micro-organisms, least harmful in a rainy season.

Testing of Drains.—It is advisable and necessary during the occupation of a house, to have the drain connections periodically tested in respect of their integrity. Systematic attention to this will often avert illnesses in a family. Whenever a house has been built, and before the drainage contract is taken from the hands of the contractor as being satisfactory, the whole plumbing and sanitary work should be tested. To this, no proper tradesman can offer any objection. It is to be hoped, also, that the time is near at hand when every householder, before occupying a house, will deem it necessary to have a certificate of its sanitary competency given at the hands of a competent inspector.

The mode of drain-testing resolves itself into one of three ways, viz.: (1) by water, (2) by smoke, or (3) by some volatile odorous substance. The water-test consist in stopping the waste and soil-pipes near the basement and each branch fitting with watertight, solid plugs, and then in filling, from the topmost part, the whole piping with water. Any imperfect joint will be revealed by leakage, and by the lowering of the original level of the water. This plan is only fitted for new buildings before occupation. The smoke-test consists in filling the whole sanitary apparatus with strong-smelling smoke under some degree of pressure. To do this, the tops of the waste-pipe and soil-pipe must be properly closed. The smoke is then driven into the pipes at the ventilating "eye" of the intercepting trap, at the junction of soil-pipe and house drain. Should leaky joints be present, they are detected by the appearance of smoke, and by its odour. In this way "faults" are tracked, and remedies applied. The apparatus used is called a smoke-testing or

Fig 45.—Fyfe and Dobson's Smoke-testing Apparatus. The arrow indicates the in-going water, which sets the smoke in motion.

fumigating machine, and consists of the following essential parts, viz.: (1) the motor by which the smoke is impelled; (2) the smoke-generating receptacle; and (3) rubber-tubing to connect Nos. 1 and 2, and No. 2 with trap. The motive power may consist of a fan driven by hand, or of spirting jets of water. Fig. 45 represents a machine of the latter form invented by the chief sanitary inspectors of Glasgow and mid-Lanarkshire. Such an apparatus works automatically when connected with a water-supply pipe. The water-tap is connected by tubing with the branch-pipe. When the jet of water enters the top-shaped part, it is split into a large series of fine sprays, which cause a current of air to pass on towards the pipe, and air passes from the outside as shown by the top arrow, to fill its place. This is a very ingenious and labour-saving machine. Other modes are absolutely useless.

Sewer Ventilation.—This subject may be briefly dis-

Fif. 46.—Drain-plan of Villa.

missed in the following sentences: By means of manholes, about every hundred yards apart, situated in the centre of the street, an interchange is effected between the air of the sewer and the atmosphere. Some of these manholes act as outlets of foul air, others as inlets of fresh, the incidence of their action depending on local wind currents, or the admission of hot water or spent steam from public works into the sewer. Such, however, at the best is but haphazard ventilation, and, generally speaking, too little attention is given to the subject. Doubtless the time will come when the sewer system will be composed of a closed system of channels, made to converge at fixed points, at which, by the exhaust power of a furnace burning at the base of a tall chimney, perfect ventilation will not only be effected, but also harmful sewer products be made innocuous.

Plan of Drains.—An exact plan of the drainage system of every house should be in the possession of its owner. From the want of such, considerable expense and trouble have been caused. To the uninitiated such a plan may appear a very complicated affair, but usually it is easier to read than an architectural plan. Fig. 46 shows a simple plan.

Disinfection of Drains.—It is by no means a simple matter to disinfect a house drain, much less a sewer. The prevalent habit of adding small quantities of disinfectants from time to time is only delusive, since, owing to the dilution which they undergo, they become practically inoperative. The only approximately efficient way is to fill a bath with water, then to charge it with a concentrated solution of some non-corrosive disinfectant, such as Jeyes' Fluid or Sanitas, mix the contents, and then suddenly discharge it by the waste-pipe into the drain. It ought always to be remembered that certain disinfectants, when used too strong, such as corrosive sublimate or chlorinated lime, attack the metal of the pipes, the former especially acting as a solvent of the solder. But of this more will be said when dealing with the disinfection of infective discharges.

### Hints to Nurses Going to India.

From a Fellow Nurse.

To nurses going out to India this autumn, a few hints for their comfort *en voyage*, borrowed from considerable personal experience in such journeys, may be acceptable.

To begin with, for the voyage pockets to hang on the berth are very useful; they are made of holland or some other strong stuff, with compartments. These do for brushes, handkerchiefs, smelling salts, lavender water, and the odds and ends one wants. One's skin gets rough; lanoline or glycerine and cucumber are useful, and marine soap is also very comforting, as it will lather with salt water, and other soap won't.

It is as well to have a deck-chair, on which have your name painted; the folding-up ones, which cost about 2s. 6d. or 3s. 6d., are best; these can be got at Curtis and Sons, Southampton, and they will do for your bed-room afterwards. They won't allow the deck-chairs that do not fold up on board.

Then at Curtis and Sons, or any other man who supplies trunks, you can get a most useful article for travelling with, *i.e.*, a brown canvas soiled-linen bag (mine has a lock and key). They cost from 4s. 6d. upwards; you can, when empty, fold them up with your rugs. I find mine useful in England.

For the cabin don't think it necessary to get the regulation cabin trunk, which is a very expensive, cumbersome article, two portmanteaux or Gladstone bags are more convenient, or say one Gladstone bag and a tin cabin trunk; these last cost about 8s. 6d. These cheap cabin trunks are much lighter, and answer the purpose as well. Anyone who has travelled much with a large leather or a large other cabin trunk knows the struggle and the bother of dragging it from under the berth, and the last straw when, hot and tired, she is diving into the depths, the lid comes down with a sudden crash right across the top of the skull. That is why a portmanteau or Gladstone bag is so handy—you can open it on the berth.

Most people who go to sea suffer for a short time from *mal de mer*; some people never get used to the sea. It is as well to be prepared for such a misfortune. I will give you here a prescription, which is supposed to be a preventative if taken regularly for a week before sailing, three times a day. I always forget to take it on land, so cannot vouch for the effect of taking it beforehand; but I find it excellent, when coming events cast their shadows before, while on board ship. I have mine flavoured with chloroform, but I give you the prescription as I received it: Bromide of sodium, two ounces; distilled water, sixteen ounces; one tablespoonful three times a day before starting. Chemists tell me this is very strong, but I have known people who have taken it double the strength, and no bad consequences have ensued. As exercise is limited on bord ship, one suffers more or less from indigestion and other internal troubles. I recommend a bottle of Burroughes and Wellcome's soda mint tabloids; they are excellent things, and very useful in India, for in the hot weather distension after food is very common. Also I recommend a bottle of Cascara Sagrada tabloids. Quinine is another drug one should never be without.

For the sake of your cabin companion, if you are a bad sailor, have the decency, if you can possibly help yourself, not to be sick in your cabin. I am one of the worst sailors ever made, and I have always been able to manage not to be ill there; you have nearly always time to get to the lavatories.

There are many ways you can practise little acts of unselfishness on board ship. The cabins are very small and stuffy, and the port-holes have often to be closed. There may be two, or even three occupying a cabin. One way you can help your cabin companions a great deal by is by not lying down in the cabin during the day; it exhausts the air so for your fellow-travellers. If you want a siesta, there is a lounge all round the dining-saloon; it is fresh and airy, and if you have your pillow you can stretch out behind one of the tables and no one be a bit the wiser you are there, and sleep comfortably. Also when the nights are hot, you can sleep up in the music saloon; it is reserved for the ladies from 10.30 p.m. It is as well to arrange with another lady to go up with you, as it is rather an ordeal to run the gauntlet of the bar alone. It is a great advantage to take a packet of your own tea. Ship's tea is never good, and if you have your own little tea-pot, one of the stewards will gladly make it for you. A box of biscuits is very nice; but you can nearly always buy one on board.

When you land in India, remember that the sun is very powerful, and avoid going about in the middle of the day, and take an umbrella with you. If you have any old umbrellas or parasols with good frames take them with you; you can get them covered for very little in India, and there is no need to take smart parasols out. I should advise your making, if you have time, one or two loose white covers for your umbrella or large parasol if you have one. You make a loop at the end of each seam to fasten the points; you make the loose cover the exact size, only a little larger, of the parasol it has to fit—it will be quickly done with the machine—put it on when the parasol is closed, and then open it up. Two frills round are pretty. Everyone uses washing covers in India, and they save your parasols. A big white sailor's hat would be as useful as anything for the voyage after Malta, and whatever winter hat you have going will do before. Not many of the lady passengers are visible before Gibraltar.

Another thing on board ship is to mind your own business, and do not interfere with anybody else's. If people quarrel—which, alas! they do—do not listen to either side, or, rather, do not take a side or appear to notice what goes on; and do not repeat things, you may make others needlessly unhappy by so doing.

I have just a little more advice to give, and that is, when you arrive in India, and live there, make your work your first object. It is a grand work, and the limit of your womanly influence is unknown. An unmarried soldier's life in India is to some extent a hard one; his life is unnatural, he is never brought under any domestic influence, and when ill and brought into wards, for the first time perhaps for many years he comes into a womanly atmosphere and under a good woman's care and influence, and no one respects a good, kindly woman like a soldier does, or appreciates so much what she does for him or the refined way she does it. To these men, for the time being, remember you are representatives of your order as gentlewomen, your profession as nurses, and your sex as women—and a soldier has a high ideal standard for all three.

### Nurses in 1896—Their Quarters, Hours, and Food.

### ST. GEORGE'S HOSPITAL.

### I.—Terms of Training.

Probationers are received at St. George's Hospital for a term of three years' training, candidates being expected to enter for a trial of two months before they are passed by the Nursing Committee. They must be between the ages of twenty-five and thirty years. At the end of two years' training, if otherwise eligible, they may be promoted to be staff nurses, being required first to pass a special examination. No nurse is promoted to the post of ward sister until she has completed the full term of three years for which she signs. Courses of lectures are given during the term of training, and classes on practical nursing are held by some of the medical officers and by Mrs. Coster, the superintendent of nurses. Examinations both practical and theoretical are held periodically. In the examination which probationers are expected to pass before appointment as staff nurse, besides the marks assigned by the medical and surgical examiner, a report is made to the committee by the superintendent of nurses as to general conduct and efficiency, and a certificate is given according to merit. A curious notice is attached to the regulations for probationers, to the effect that candidates must be not less than 5 ft. 3in. or more than 5ft. 6 in. in height.

### II.—Hours of Work and Times off Duty.

The number of working hours are variable, the daily hours off duty ranging from two to four and a half. The actual hours in the wards alternate between 9½ and 11½. Nurses come on duty at 7 a.m. and leave the wards at night at 9.30; during these hours they are off duty every day from two and a half to four and a half hours (including time for one meal), besides being absent from the wards for their other meals. Hours off duty for nurses and probationers are from 7 to 11 a.m., 10 a.m. to 1 p.m., 2 to 6 p.m., and 6 p.m. to 10 p.m. When off in the early morning nurses are expected to be present at breakfast. When leave is from 10 to 1, time for dinner is included, and for the 6 to 10 leave, tea being at 5.30, four and a half hours are practically obtained away from the wards. Sisters come on duty an hour later than the nurses and probationers, and are off duty alternately an hour and a half one day and four hours the next. Night nurses are on duty from 9.30 p.m. to 9 am., going out either before or after bedtime. Seven hours is about the time actually allowed for sleep for day and night staff. The time table appears to be a very elastic one at St. George's. The details are not printed, but written rules hang in the dining-room subject to alterations. None of the staff are permitted to sleep away from the hospital on days off. Every nurse has a day off once a month, and these dates may be booked weeks in advance, with the view of allowing a convenient choice of dates. Late leave may be had with reasonable frequency, and theatre tickets very often find their way to Mrs. Coster's hands through the generosity of theatre managers. A list is kept, and these pleasures are distributed in rotation amongst the staff. Nurses and probationers have a fortnight's holiday in the year; sisters a month.

### III.—Meals.

A housekeeper is responsible for the meals of the nursing staff, under the supervision of the matron, and presides over them. The day nurses breakfast at 6.30 a.m., having tea and coffee, bacon, eggs, and bread and butter. For dinner hot meat, always two vegetables, and pudding is provided. Tea is served in the dining-room in the afternoon, and supper, consisting of fish, cold or warmed-up meat, or cheese, with milk or beer, as at dinner, is served at 9.30. No food except the night nurses' meal and the early lunch are taken out of the dining-room. For these the night nurses take their allowances for the night away with them from breakfast at 9 p.m., consisting of eggs, fish, or cold meat, and the day nurses fetch their milk and bread and butter from the dining-room in the morning. These meals are taken in the ward or ward kitchen. Sisters still dine at the old-fashioned hour of four o'clock, but if off duty in the afternoon are not obliged to be present at this meal.

### IV.—Salaries and Uniform.

Probationers during their first year are paid £10, £14 for the second, and for the third, if not promoted to be staff nurses, £20. Staff nurses' salaries begin at £22, rising to £30; sisters begin at £36, rising to £40.

Indoor uniform is provided by the hospital, two dresses of black Victoria cord being given to the sisters each year, while the nurses and probationers are provided yearly with three galatea dresses; aprons and caps are also given as required. An allowance of £4 a year is allowed for washing to each of the nursing staff. All the staff are required to wear outdoor uniform, except on their monthly and annual holidays, and this they are required to provide for themselves.

### V.—Nurses' Quarters.

The quarters provided for the nursing staff at St. George's Hospital are on the top floor of the hospital, the ward sisters having their bed and sitting rooms (the former leading from the latter by a little staircase) off their wards. The cubicles allotted to the nurses and probationers are of a fair size, each with a window, and the dormitories are light and airy, the little apartments being comfortably furnished. Some of them command a fine view over the parks. It is in sitting and dining-room space that the accommodation at St. George's is deficient. There is but one dining-room for the whole staff, and that is in the basement, entered from the corridor sacred for the most part to porters and servants. Here, too, is the Matron's office—a miserably small room for the busy head of a large training school—with no ante-room, so that those wishing to see the Matron on business have to stand in the aforesaid dark and draughty passage. There is no sick room for the nurses at present; they are sent to the wards when ill. In the new home now being built, at some little distance from the hospital, separate bed-rooms are to be provided for the nurses, and sitting-rooms and a sick-room are to be included. The night nurses' cubicles are together in a separate dormitory, under charge of the night superintendent, who sees that the door of communication is locked when the nurses are in bed, to prevent possibility of disturbance.

### Minor Appointments.

Middlesex County Asylum, Wandsworth.—Nurse Jane Elizabeth Wilde has been placed in charge of the infirmary ward at this asylum. Nurse Wilde received her training at the Wandsworth and Clapham Infirmary.

Perth District Asylum, Murthly, N.B.—Nurse Berwick has been appointed Charge-Nurse at this asylum. She received her training in general nursing at the Edinburgh Royal Infirmary, and has also worked at the Royal Edinburgh Hospital for Sick Children.

Swansea General Hospital.—Miss Amy M. Yarrow has been appointed to the post of Sister at the Swansea Hospital. Miss Yarrow was trained at the Royal Southern Hospital, Liverpool, and has worked as charge nurse at the Ellesmere Port Hospital, Manchester Ship Canal, and the South-Eastern Fever Hospital, London, whilst she has also had charge of the out-patient department, Royal South Hants Infirmary, Southampton, and held the position of sister-in-charge at the Royal Stanley Hospital, Liverpool.

### Midwifery Papers.

### VI.—MANAGEMENT OF NATURAL LABOUR.

When a nurse is called to a confinement case she should convince herself by examination that labour has commenced. In highly nervous women, and frequently in first cases, false labour pains occur some days, it may be, before the confinement comes off, and are mistaken for true pains. False pains are generally felt in the front of the abdomen; they do not affect the dilatation of the os or accelerate the labour. They are often induced by a loaded condition of the bowels, and disappear when an enema has been given and the bowels relieved.

The signs of labour are the onset of true pains, or regular painful uterine contractions; a discharge of mucus tinged with blood, called the "show"; softening and shortening of the cervix with dilatation of the os-uteri, and the protrusion of the amniotic-bag. The regular uterine contraction can be distinguished abdominally by placing one's hand over the fundus, when the uterus can be felt to harden and relax at intervals. The nurse should then make a vaginal examination. Before examining her patient the nurse, having washed her hands (using a nail-brush), and dipped them in $\frac{1}{1000}$ sol. of perchloride of mercury, should sponge the external genitals, then lubricating the first finger of her right hand with vaseline, she should pass it into the vagina, feeling gently upwards till the cervix is reached. In the early stage of labour the cervix is high up in the vagina, and is rather difficult to reach. During this examination the nurse should notice the extent of softening and dilatation of the cervix, and that the presentation is normal (the hard head of the child is found low in the uterus), and that there are no abnormal conditions, such as growths or contractions, present in the pelvis or vagina to prevent natural labour. The greatest gentleness should be used in making a vaginal examination, so as not to injure the soft parts, and if the os is sufficiently dilated to enable the membranes to be felt, care should be taken not to prematurely rupture them, for their early rupture would be dangerous to both mother and child by causing delay of birth, and so increasing the risk of hæmorrhage. It is enough for the nurse to know that the presentation is normal, that the labour has begun, and that there is no reason why it should not progress favourably.

During the first stage the patient's bowels and bladder should be emptied. It is, perhaps, always well to give a simple enema of soap and water, as patients very often tell one the bowels have acted when the motion has been very slight, and a large constipated mass has been found later on impeding the labour. During the intervals of the "pains" the patient should be encouraged to move about the room, and beef-tea, or a cup of tea, or any other light nourishment should be given. Alcohol should never be given except by special medical order, as it increases the risk of hæmorrhage. The patient should be undressed, and her petticoat and night-dress arranged; a warm shawl or dressing-gown should be worn over her night-dress while she is sitting up. Towards the end of the first stage the patient should be placed in bed, and a hot douche (110 deg.) of Condy and water should be given to soften and cleanse the parts. The nurse should examine at intervals to note the progress of the labour.

When the second stage commences, after the rupture of the membranes, the nurse should note if there is any rigidity of the perineum. If so, it is useful to foment it frequently with hot water, and to rub in olive oil. In fomenting use absorbent wool instead of a sponge, as it can be easily burnt and replaced when soiled. Towards the end of this stage, when the perineum is much distended, it should be supported by placing the finger and thumb of the right hand each side of the fourchette, the hand behind pressing it slightly forwards to prevent laceration. When "bearing-down" pains set in the patient can often assist by voluntary muscular effort, but she should not be allowed to over-exert herself and waste her strength if the labour is slow. In this stage she finds it an assistance to pull on a jack-towel secured at the foot of the bed, but this should not be allowed when the pains are strong and the presenting part is advancing rapidly, and never in cases of first confinements, as a too hurried emptying and contraction of the uterus may result in that terrible fatality—rupture. When the labour is at its height, the pains strong, and the perineum much distended, the patient should be encouraged to cry out, so as to relax the muscles. If the child's head advances too rapidly, delay it slightly, and equalise the pressure on the vulval outlet by pushing the head gently towards the pubic arch. During this stage the left hand should be placed upon the patient's abdomen, the fundus of the uterus should be grasped, which should be followed down as it descends in the pelvis. Grasping the fundus is important as it stimulates uterine contraction and is a safeguard against hæmorrhage, and on no account should the grasp be relaxed until the labour is complete. The head of the child must be received by the midwife's right hand, and guided towards the mother's thigh as the trunk is expelled. As soon as the head has passed the vulva, feel if there is a loop of cord round the neck; if this is so, slip it over the head if possible, if not push it as far as you can over the shoulder. Directly the child is born wipe the eyes (if possible before the lids open) with some clean, soft rags, which should be in readiness. If the child does not cry of itself immediately after birth it must be made to do so by briskly rubbing up and down the spine, or by flicking its chest and back with a damp towel. The mouth and nose should be wiped out with the soft rag to clear them from mucous. When the veins in the cord have ceased pulsating, it should be tied with strong silk or waxed thread, about four inches from the umbilicus, and again a second time a couple of inches lower down. The cord should then be cut between the two tied portions. A blunt-pointed scissors, which has been dipped in perchloride of mercury $\frac{1}{1000}$ should be used. Of course, the rag, scissors, and silk should all be ready to hand, together with a little flannel shawl to wrap round the baby, which should be removed from the bed after the cord is cut, and placed in the cradle till it can be attended to. All this is done by an attendant, who should assist, when the second stage has begun, to support the patient's right thigh if required. The midwife should direct the assistant, but should not relax her grasp of the uterus until the placenta is expressed at the end of the third stage by external pressure. This is done about 15 to 20 minutes after the birth of the child, and during this interval the thrombi form in the veins. Advantage should then be taken of an active contraction of the uterus to express the placenta. This is done by pressing hard on the fundus with the palm of the hand, and relaxing and contracting the pressure of the fingers. If there is much hæmorrhage after the birth of the child, express the placenta at once to allow the uterus to contract. Carefully examine the placenta afterwards to see that no portion of it or shreds of membrane are left behind. Examine the perineum to see that there is no laceration. Give a hot Condy douche (110 deg.). Apply a tight binder and place a pad of wood wool to the vulva to receive the discharge about twenty minutes or half hour after the placenta is expressed if there is no hæmorrhage. Give a fluid drachm of ergot in a little water immediately after the placenta is born, but never before. Ergot is given to excite contraction of the uterus, and it is unsafe to give it at any time during the labour until the completion of the third stage. In giving a douche never fail to test its heat with the bath thermometer, and use, if an irrigator is not available, a Higginson's syringe fitted with a glass tube. Nurses need not be reminded that these articles should be perfectly clean and aseptic. The pressure on the fundus should not be relaxed when the douche is given after labour, but the water of the injection should be expressed as it enters the womb. Care should be taken to give this douche gently, and to fully expel all air from the glass tube before it is inserted in the vagina, as water or air getting into the open blood-vessels would be a serious danger to the patient. When the nurse has convinced herself that there is no hæmorrhage, the soiled draw-sheet and mackintosh should be removed from the bed, and the petticoat slipped off, and the patient's night-dress unrolled. A little warm milk or gruel may now be given in a feeder, and the patient should be allowed to rest and sleep if possible. The pillow should be low. The pulse and temperature should be taken after labour. If the pulse is 100 or more great watchfulness is necessary, as there is danger of hæmorrhage. The pad should be examined to notice discharge, and the contraction of the uterus should be noticed. Immediately after the labour the uterus should contract into a hard mass the size of a cricket-ball. After the baby is washed and dressed, it should be brought to the mother and placed to the breast, and allowed to suck for a short time, as this encourages uterine contraction.

### Novelties for Nurses.

### WOOLLEN GARMENTS.

There is always a satisfaction in getting goods direct from the manufacturers, as it is the best way to secure the same quality and make which has met with ourapproval on a previous occasion. Intending purchasers of warm clothing will do well to send for the catalogue of the Knitted Corset and Clothing Company, 118, Mansfield Road, Nottingham. It contains illustrations of garments and samples of the materials in which they can be bought. Purchasers to the value of £1 are encouraged by the presentation of a nice little woollen cape, a much more suitable form of wrapper than a shawl, as it does not fall off the shoulders, and looks besides neat and tidy. These little capes form very useful Christmas presents for chilly people.

### EGERTON BURNETT, LIMITED.

The cold and unsettled weather which has procured for the latter part of the summer of 1896 an unenviable notoriety shows no signs of amendment, and the swiftly approaching days of "chill October" suggest the addition of sundry warm articles of clothing to the wardrobe. The thoughts fly naturally to tweeds and serges, and Mr. Egerton Burnett is, as usual, to the front with a selection that it would be impossible to surpass and difficult to equal. The most fascinating designs appeal to lovers of fashion, though for good taste, durability, and comfort there is nothing to come up to the Royal Navy serge. It defies alike rain, salt-water, or sun, and preserves a good appearance to the end. Of coloured serges and homespuns there is a large variety which would serve a number of useful purposes. All these goods are manufactured of pure wool, and are thoroughly to be relied on. For cycling purposes they will be found admirable, as they are both warm and light, and the cost is so reasonable as to bring them within range of the most limited means. A white Scotch winsey calls for special mention. It is unshrinkable, and from its extra wide width and softness of texture is eminently adapted for under-clothing. For those who are prohibited from sleeping in linen garments it would make charming nightdresses, and will be found specially useful for babies and young children. There is also an excellent waterproof cloaking in very fine twill serge, navy or black, which nurses would delight in for summer wear, and which in selection could be made up into whatever shaped cloak they might desire. Mr. Egerton Burnett, however, does not confine his assortment to woollen materials alone. Cambrics and cottons are a special line calling forth our warmest approbation. The grey and blue zephyrs, and are to be had in every shade. There are also a variety of ingrain materials that will appeal to the heads of establishments who are considering the difficult problem of uniform. All these that we mention are fast colours, and guaranteed to stand any amount of washing.

Travelling rugs are another specialité of this enterprising house. The Royal Wellington rug is greatly to be desired, both on account of its warmth and lightness, as well as its convenient and ample size. Austrian blankets with pretty fancy stripes are also in evidence, and, in conclusion, we desire to draw our readers' attention to the excellent tailoring department, recently started in connection with the firm. Plain skirts for walking or cycling can be made to measure from any material that is selected, and as the best workmanship only is employed, every satisfaction is given to those who avail themselves of this convenience. Self-measurement forms, patterns, and tailoring price list can be had on application, post free. Our readers cannot do better than make an early application before the busy season commences.

### COCOA AND CHOCOLATE.

**Fry's chocolate is almost a household word, so much so that it would seem almost unnecessary to call so familiar a subject to the notice of our readers. Yet the love of novelty is a powerful factor in leading people from a well-tried and trusted friend. Some imagine that the very best chocolate and cocoa is not obtainable from our English manufacturers, and they pay a large price for foreign goods which they could obtain for a moderate sum by purchasing home products of the best kind. Messrs. Fry and Sons have magnificent machinery at command, and apply the highest scientific knowledge in manufacturing from the very best materials. The consequence is that they can now compete with any chocolate makers in the world, and are able to guarantee the purity of their goods besides. We know no better cocoa than their pure concentrated soluble cocoa. It is delicious to the taste, and economical in use. The flavour of the nut is most agreeable. A most excellent chocolate is secured by the use of the "Ceylon" chocolate, which is flavoured with vanilla, and is a very nice sweetmeat also. The Caracas chocolate, too, is first-rate for both purposes, and we can confidently say that those who give the cocoa and chocolates we mention a trial in their households will be not likely to return to the use of foreign products. Messrs. Fry make fancy bon-bons also of great variety, amongst the best of these being the chocolate almonds, walnut fouré, and cream almonds. The dessert chocolate in floral desfgns is most excellent.**

### A Nurses' Friend.

Miss Eleanor Smith (a sister of the late Professor Henry Smith), who has just died at Oxford, was well known in that city in connection with nursing matters. She was a member of the Committee of Management of the Radcliffe Infirmary, and was keenly interested in the Sarah Acland Home for Nurses. In the days before the Sarah Acland Home was founded Miss Smith herself started and maintained a district nurse in Oxford; indeed this scheme of hers was the foundation from which this institution took its rise.

### A Book and its Story.

"MAGGIE."*

In the compass of a small volume Mr. Stephen Crane gives us a strangely full and complete picture of one particular phase of human life—gutter life, to which the characters in his book were born, and out of which they never rise. Maggie tried to rise, but circumstances were against her. From her conditions "it all had to be, and these were the conditions." Her story is no new one, and there lies its tragedy, in its dominating and fatal necessity. Instances of a like nature constitute the past history of all poverty-stricken city life, but Mr. Crane's pen has somehow brought it all a little more vividly before us than other pens have done. "Rum Alley" exists for us as we turn its pages, and its inhabitants are actualities. Here we are concerned with the Johnson family, a father, a mother, and three small children, named respectively Maggie, Jimmie, and Tommie. The personality of the last-mentioned does not monopolise much interest; his existence terminates in Chapter IV. "The babe, Tommie, died. He went away in an insignificant coffin, his small waxen hand clutching a flower that the girl, Maggie, had stolen from an Italian."

She and Jimmie lived. The inexperienced fibres of the boy's eyes are described as being "hardened at an early age. He lived some red years without labouring. During that time his sneer became chronic. He studied human nature in the gutter, and found it no worse than he thought he had reason to believe it. He never conceived a respect for the world, because he had begun with no idols that it had smashed. . . . About this time his sneer grew so that it turned its glare upon all things. He became so sharp that he believed in nothing. To him the police were always actuated by malignant impulses, and the rest of the world was composed, for the most part, of despicable creatures who were all trying to take advantage of him, and with whom, in defence, he was obliged to quarrel on all possible occasions. He himself occupied a downtrodden position, which had a private, but distinct element of grandeur in its isolation."

Jimmie's mother is described in the same inimitable, terse, and suggestive manner. A woman of ungoverned temper, rendered all the worse by constant fits of drunkenness, she is pictured to us as drinking all the morning, and breaking such of the furniture of the garret as is not previously broken, all the afternoon. In such surroundings Maggie and her brother passed the first tender years of their lives. The girl, Maggie, blossomed in a mud puddle. "She grew to be a most rare and wonderful production of a tenement district, a pretty girl." None of the dirt of the New York Rum Alley seemed to be in her veins. When a child, playing and fighting with gamins in the street, "dirt disgusted her. Attired in tatters and grime, she went unseen."

As she grew older her looks improved. At last Jimmie, who by now was working himself, reminded her she must do the same. The girl got a position in an establishment where collars and cuffs were made. "She received a stool and a machine in a room where sat twenty girls of various shades of yellow discontent."

About this time the head of the Rum Alley family died, and Jimmie grew large enough, as Mr. Crane puts it, "to take the vague position of head of the family." As incumbent of that office, he stumbled up late at night as his father had done before him. "He reeled about the room, swearing at his relations, or went to sleep on the floor."

And the mother of this young hopeful had by now risen to such a degree of fame she could "bandy words with her acquaintances among the police-justices. Court officials called her by her first name. She measured time by means of sprees, and was eternally swollen and dishevelled."

Maggie's squalid romance commenced with the entrance of a certain young man named Pete, who had formerly lived in a neighbouring alley. One evening he called to see his former companion, Jimmie, at the Johnson home.

Maggie watched Pete.

"He sat on a table, and dangled his checked legs with an enticing nonchalance. His hair was curled down over his forehead. His blue, double-breasted coat, edged with black braid, was buttoned close to a red puff tie, and his patent leather shoes looked like weapons." The description is life-like of this young dandy, who is to exert so pitiful an influence on the girl who watched him admiringly. To her, his manœuvres stamped him as a man who had a correct sense of his personal superiority. For Pete was superior; there was no question of that.

Maggie, as has been said, was pretty, with a fresh child-like prettiness, out of keeping with her surroundings. The tawdry-dressed Pete was a vision out of another world; he, in his turn, admired the girl.

Maggie's head swam with pride. She reflected sadly on the contrast in their surroundings; she imagined the young man rich, whilst to her "the earth was composed of hardship and insults."

Pete further impressed Maggie with his magnificence, when he took her out of an evening to places of amusement, at which entertainments he displayed a complete unconcern, the nonchalance of custom the girl argued in language of her own.

"Maggie was anxious for a friend to whom she could talk about Pete. She would have liked to discuss his amiable mannerisms with a reliable mutual friend. At home, she found her mother often drunk, and always raving. It seemed that the world had treated this woman very badly, and she took a deep revenge upon such portions of it as came within her reach. She broke furniture as if she were at last getting her rights. She swelled with virtuous indignation as she carried the lighter articles of household use, one by one, under the shadows of the three gilt balls, where Hebrews chained them with chains of interest."

One evening, returning home, the Mother Johnson greeted Maggie even less amiably than usual. The truth was not far to seek. The room was a débris of broken crockery. A volley of oaths greeted the girl. Jimmie and his mother had not confined themselves to articulate language, but had expressed themselves in a more forcible way. Maggie had entered the scene immediately after a fight between the two. Pete was with her.

"The woman on the floor cursed. Jimmie was intent upon his bruised forearms." Mrs. Johnson from the floor lay and swore at her daughter. Her hearer shuddered—the woman's violence increased—"Git th' devil auta here," she shrieked. Pete seized his opportunity—and Maggie went.

The closing scene in Mr. Crane's book shows us in an unrivalled manner his consummate art in the grasp of a situation, and leaves the only impression of mère Johnson which we carry away free of disgust. Some months after the elegant dandy had carried off her daughter, news is brought the mother of her Maggie's death. "Yer poor misguided chil' is gone now, Mary, an' let us hope its fer deh bes'," the neighbour says. "Yeh'll fergive her now, Mary, won't yehs, dear, all her disobed'ence? All her t'ankless behaviour to her mudder an' all her badness? She's gone where her ter'ble sins will be judged. Yeh'll fergive her, Mary?" pleaded the woman. The mourner essayed to speak, but her voice gave way. She shook her great shoulders frantically in an agony of grief. Finally her voice came—"Oh, yes, I'll fergive her! I'll fergive her."

* "Maggie." By Stephen Crane. London: William Heinemann, 1896.

### Dress and Uniforms.

By a Matron and Superintendent of Nurses.

### ENGLISH NURSES IN SHANGHAI.

The want of trained nurses to attend on the sick in their own homes has been sorely felt for some time past in Shanghai. Efforts have been made periodically to meet the difficulty, but hitherto without success. The Municipal Council have now, with approval of the ratepayers, taken the matter in hand, and formed a committee to establish on a firm basis a small institution which in time, it is hoped, will be self-supporting, but the initial expenses of which will be defrayed out of public funds. The head nurse is to receive a salary of £150 per annum, and the nurses £100 per annum; they will be provided, in addition, with board, lodging, uniform, and washing. The nurses are to be engaged for not less than three or more than five years, and all travelling expenses, including first-class passage from London to Shanghai, will be paid by the Council. The illustration depicts the three nurses who are going out to pioneer this admirable scheme. Miss Campbell, who has been selected as head nurse, is shown on the left, wearing the tasteful uniform adopted by the institute. It consists of a grey beige dress, made quite plain, with full sleeves gathered in at the wrist under a neat white cuff. The apron is made of the finest

HEAD NURSE — OUT DOOR UNIFORM — NURSE

linen, with a high bib up to the throat, and straps crossing behind, and fastening at the waist. The cap is a charming arrangement of white Indian muslin, about a yard square, which is folded triangularly, and rolled so as to form a coronet round the face, and caught into position by a stitch or two at the back, leaving a long, graceful end, which falls over the hair and reaches as far as the waist. The nurse's costume differs only in the dress, which is a very pretty blue and white striped zephyr. It has the merit of being cool, as well as serviceable in colour, for Shanghai is subjected to extremes of heat and cold, which make it necessary to provide a wardrobe accordingly. The central figure of the group shows the outdoor uniform, which in summer consists of a dress of grey zephyr, a thin grey cloak, and grey bonnet and veil. In winter the dress is grey beige, and a warm, comfortably lined cloak of grey homespun is worn as an outer covering. The Council are fortunate in having secured the services of such able women as Miss Campbell, Miss Low, and Miss Gadwell. Miss Campbell was trained at Winchester, and from thence went to Southampton as sister, and lastly to Lewisham Infirmary as assistant matron, which post she filled with consistent tact and dignity. Miss Low was trained at the West London Hospital and the Clapham Maternity Home, after which she was appointed staff nurse at the Lewisham Infirmary. She has recently had valuable experience in private nursing as a member of the Nurses' Co-operation in New Cavendish Street. Miss Gadwell received her training at King's College Hospital, and since then has worked on the private nursing staff. If skill, ability, intelligence, and zeal are the important factors we have always understood them to be in the achievement of success, then, indeed, the Shanghai Nursing Institution has nothing to fear for the future, and has every cause for congratulation in the present.

### Mediæval Practice in the Care of the Sick.

It is said that in Japan, when a rich man would make a present to his friend, he sends with his costly gifts a small piece of dried fish in token of humility.

At the last International Congress of Hygiene held at Buda Pesth, a valuable paper was read upon "The Progress of Nursing in the British Isles," signed by Her Royal Highness the Princess Christian of Schleswig-Holstein, in her capacity as president of the first corporation of women which has received the privilege of a Royal Charter.

The method and precision displayed during recent years in the scientific training and organisation of educated women as nurses for the sick; the encouragement and patronage bestowed upon the profession of nursing by Her Majesty the Queen, and in some measure also by almost every member of the Royal family, have enabled English hospitals, training schools, and nursing associations to rank before any kindred institutions in Europe; and the inestimable benefits conferred upon the sick by their means may be compared to the valuable offerings the Japanese bestows upon his friend.

But the accompanying morsel of dried fish is intended to remind prosperous inhabitants of Japan that their ancestors were only a race of poor fishermen, and that pride ill becomes persons of such humble origin.

Many friends of modern nurses are fearful lest in the glory of "Chartered corporations," and stiff examinations after the advanced instruction given to them in the schools upon anatomy and physiology, they may be inclined to imitate that conduct complained of by an American preacher, who said "Some sisters amongst us have cut their tiller ropes and gone it with a rush," and by invading the province of medical men, forget the womanly qualities of tenderness, sympathy, and patience with human weakness, proper to their sex.

The future of the ever-increasing army of trained nurses we are not competent to foretell, but even as the Japanese morsel of dried fish, we venture to offer these few thoughts and facts concerning the humble genesis of the nursing profession in what are called the dark ages; hoping, indeed, that the brilliance of modern attainments will be enhanced by the contrast; but, also, that something more than a pitying smile at their ignorance may be bestowed upon the women who gave of their best to aid the sick in days when charity was, perforce, of greater account in the world than scientific training.

In considering the subject of mediæval nursing, it must be borne in mind that no such thing as a "profession" of the art existed; nevertheless, the practice of nursing must necessarily be carried on, skilfully or unskilfully, wherever there are sick people, kind hearts and willing hands, or physicians to give instructions regarding their patients.

We have, in fact, a tolerably large collection of medical works preserved to us, extending from Saxon down to Stuart times, which give detailed directions for the treatment of patients in respect to dressings for wounds, baths, particular forms of bed making, dieting, and other matters which must have fallen within the province of the nursing as distinct from the medical art, many of which directions it is obvious could hardly have been carried out by the physicians themselves. Much incidental light is thrown upon the manners and customs of our ancestors by these instructions for nurses, but as a rule they are only to be found buried amongst a mass of old-world medical lore, interesting only to the historians of scientific progress or to the medical antiquarian, consequently few people are acquainted with even the names of the books or their dry-as-dust authors.

Here and there, in the pages of romance, of the lives of saints, which correspond somewhat to our modern novels, we catch a glimpse of the sick beds of wounded knights, or of princesses dressing the sores of loathsome lepers, but there is nothing systematically recorded, and the incidental accounts are not, as a rule, detailed.

One naturally turns to the Catholic Church of to-day to find analogies for the life of our ancestors before the Reformation; but although the little company adhering to the See of Peter passed through the rugged defiles of English political life in the 16th century, they emerged into such a new country, with such new scenery, their path was beset with such different, if increased difficulties, while the horizon of their world has become of late so enlarged, that even they, looking back from the end of the 19th century, to their co-religionists of the 13th and 14th, must upon some matters say, "Your thoughts were not my thoughts, nor are your ways my ways." And the attempt to gather up the scattered fragments of information concerning the practice of nursing in the middle ages, which are preserved in the pages of our extant early English literature, serves to give one more proof of the greatness of the gulf fixed by the renaissance between the old world and the new.

### Appointments.

### MATRONS.

Florence Nightingale Hospital for Infectious Diseases, Bury.—Nurse Bradley has been appointed Nurse Matron at this hospital. Her previous experience has been gained at the City Infectious Diseases Hospital, Newcastle-on-Tyne.

Davidshill Fever Hospital, Dalsy, Ayrshire.—Miss L. A. Ediss was appointed Matron of this hospital on September 11th. She was trained at the Monsall Fever Hospital, Manchester, and has since acted as nurse matron at Lennox town Fever Hospital, Stirlingshire.

### Female Sanitary Inspectors.

The Vestry of St. George's, Southwark, have appointed Miss A. Elliott as an additional sanitary inspector for their district, subject, of course, to the approval of the Local Government Board, and we shall watch the result of the experiment with interest, not unmingled with misgiving.

The chief duty of the new inspector will be the enforcement of the bye-laws under the Public Health Act with respect to houses let in lodgings or occupied by more than one family, and there is no doubt that the places which will have to be inspected comprise some of the worst slums and alleys in London. We are far from saying that no such work should be undertaken by women. There is much that a clever woman can do by persuasion which a man might fail to obtain by threat of law, and so far as the visits by day, the inspection of the sanitary arrangements, and the explaining to the women of the place the obligations which the law imposes on them are concerned, no exception need be taken to the appointment. But the carrying out of the bye-laws involves far more than this, including, as it does, the inspection of these houses by night, and we cannot think that the vestry have acted wisely in appointing a woman to such work. The position of a sanitary inspector is very different from that of a missionary woman. The latter goes of necessity as a friend, in many cases she goes as an almoner—through her distress is brought to the notice of the charitable, and it is to the interest of the whole court or alley that she should be unmolested. The sanitary inspector, however, if she is to do good work at all, cannot act entirely by persuasion; she must at times put the law in action, she must then appear as an ally of the police, and we fear she must incur the enmity of many who on these night visits will have ample opportunity of paying her off. We hope that on these occasions she will not go about her work as an "unprotected female." Whether, however, "a night out" with the "lady inspector" will become a popular pastime with the police may perhaps be questioned, and some may even ask whether they could not "do the job just as well themselves." In any case it is far from being ideal woman's work.

### Everybody's Opinion.

[Correspondence on all subjects is invited, but we cannot in any way be responsible for the opinions expressed by our correspondent. No communication can be entertained if the name and address of the correspondent is not given, or unless one side of the paper only be written on.]

### ORPHANS CAGED.

A "Medical Correspondent" writes: I was sorry to observe, on a recent visit to St. Mary's Home, Broadstairs, (the seaside convalescent home for children, in connection with the Kilburn Sisterhood), that the infant inmates are confined at night in what are euphemistically called cubicles, but as a matter of fact bear a strong resemblance to the cages of a travelling menagerie. In strange contrast to the noble proportions of the dormitories and their uninterrupted outlook upon the expanse of ocean, the poor infants who sleep therein are "cribbed, cabined, and confined," each within its own enclosure of iron bars, from which no escape is possible till the clips which fasten the prison doors are withdrawn. It is terrible to contemplate the consequences in case of an outbreak of fire under such circumstances; no escape would be possible for the unhappy inmates of the cubicles until the doors had one by one been unfastened. Attention has already been called by The Hospital to this matter in connection with the parent institution at Kilburn; and we understand that some arrangement for the simultaneous opening of the iron doors has been adopted there. At Broadstairs, however, each cubicle must be opened singly, and how this would be done in a moment of panic it is difficult to see. It is true that the older inmates are trained in the use of fire-extinguishing apparatus, and that some would probably be charged with the duty of releasing the children. Theoretical preparation and practical accomplishment are, however, two different things; and the question which the good Sisters should consider is whether the economy of personal vigilance gained by the adoption of these cages is not dearly purchased when it entails the risk of a holocaust of innocents. Moreover, we would ask, is the substitution of such mechanical restraint for loving personal care worthy of a community of Christian workers at the end of the nineteenth century?

### UNIVERSITY COLLEGE HOSPITAL NURSES.

Sister Cecilia, sister superior, writes: I beg to call your attention to the enclosed cuttings from the Hospital Nursing Mirror for September 26th, as the article from which they are taken seems to have been chiefly compiled from papers of regulations which were in force before the alterations as to nurses' and probationers' times off duty. You will see by the corrections in red ink that each nurse and probationer now has a daily "pass" for two hours, and we shall feel obliged if you will kindly publish a fresh statement of our present regulations, as the information given in your issue of this week is misleading. The authorities will be most thankful if the public will come forward and help them with the means to build a proper nurses' home. It is true that the probationers "pay for their training," *i.e.*, £65 for the three years' course, but this does not cover the expense of their board and uniform, and of uniform and household washing.

The statement Sister Cecilia encloses is as follows:

Nurses and probationers go on day duty in the wards at half-past eight a.m.

Nurses and probationers have two hours off duty daily, and one free evening every week. The free evening is from six p.m., and the sister of the ward arranges, if possible, for the nurses and probationers, when they have their free evenings, to take their daily pass of two hours on the following day, from half-past eight to half-past ten a.m., so as to give them longer consecutive time out of the wards. Night nurses do not get up until half-past five p.m.

Nurses and probationers occasionally have a whole day off duty, by special arrangement.

Tea, coffee, and cocoa are kept in a cupboard in the ward scullery.

Tea is not taken to the nurses in the wards. Day nurses go to the dining-room for tea; the night nurses have their tea taken to their bedrooms at four p.m. because they prefer it so.

This (*i.e.*, that the hospital and private staffs are interchangeable) is a misunderstanding. The hospital nurses are not sent to private cases. The private staff rest at the home in the intervals of their cases, and they come on duty in the hospital when the ward nurses are away for their annual holidays, or in some cases of emergency, as when a hospital nurse is ill.

If incapacitated by illness or old age, hospital or private staff nurses receive a pension and are provided for.

The temporary quarters are a large house in Tavistock Square, where the nurses are very comfortable. There are only three bedrooms in which three probationers sleep, and they are very large rooms.

*** The particulars we published last week were taken from the printed rules supplied to our representative at the end of last February. It was stated expressly, as we said last week, that the hospital and private staff of ses" are not interchangeable. Sister Cecilia, in the course of the interview, stated in effect that "the nurses kept up their hospital knowledge between cases." The temporary quarters in Tavistock Square have been taken since February. We shall incorporate Sister Cecilia's statement in the notice of University Hospital when the articles on the subject are republished in book form. We are glad to see how deep and widespread is the interest excited by these articles, and to find they are having good effect in various directions by causing changes which all must cordially welcome.—Ed. *T. H.*

### For Reading to the Sick.

HIS TENDER SOLICITUDE.

**Motto.**

"Wherefore comfort one another with these words."

Mine is an unchanging love,
Higher than the heights above,
Deeper than the deeps beneath,
Free and faithful, strong as death.

—*Cowper.*

For the love of God is broader
Than the measure of man's mind;
And the Heart of the Eternal
Is most wonderfully kind.
But we make His love too narrow
By false limits of our own;
And we magnify His strictness
With a zeal He will not own.

—*Faber.*

Change and decay in all around I see,
O Thou, Who changest not, abide with me!

—*Lyte.*

Thou wert always our Father! each sun that arose
Has done nothing through life but fresh mercies disclose;
But we feel, while the joy of our life is laid low,
Thou hast ne'er been so tender a Father as now.

—*Faber.*

**Reading.**

"The very hairs of your head are all numbered."—*Matt.* x. 30.

What a word is this! All that befalls you, to the very numbering of your hairs, is known to God! Nothing can happen by accident or chance. Nothing can elude His inspection. The fall of the first leaf, the fluttering of the insect, the waving of the angel's wing, the annihilation of a world—all are equally quoted by Him. Man speaks of great things and small things—God knows no such distinction. How especially comforting to think of this tender solicitude with reference to His own covenanted people; that He metes out their joy and their sorrows! Every sweet, every bitter, is ordained by Him. Even "wearisome nights" are appointed. Not a pang I feel, not a tear I shed, but is known to Him. Every moment the everlasting arms are underneath and around me. He keeps me as the apple of His eye.

Do I look to the future? Is there much of uncertainty and mystery hanging over it; it may be of much premonitory of evil? Trust Him. All is marked out for me. "He keepeth the feet of His saints." A hair of their head will not be touched.

He leads sometimes darkly, sometimes sorrowfully; most frequently by cross and circuitous ways we ourselves would not have chosen; but always wisely, always tenderly. With all its mazy windings and turnings, its roughness and ruggedness, the believer's is not only *a* right way, but *the* right way. "Nothing," says Jeremy Taylor, "does so establish the mind amidst the rollings and turbulence of present things, as both a look above them and a look beyond them; above them to the steady and good hand by which they are ruled; and beyond them, to the sweet and beautiful end to which, by that hand,they will be brought."

"The Great Counsellor," says Thomas Brooke, "puts clouds and darkness round about Him, bidding us follow at His beck through the cloud, promising an eternal and uninterrupted sunshine on the other side." On that "other side" we shall see how every apparent rough blast has been hastening our barks nearer the desired haven. There was no redundant drop in the cup of His own sufferings; neither will there be in that of His people. "Though He slay me, yet will I trust in Him."—From "*The Mind and Words of Jesus.*"

### Notes and Queries.

The contents of the Editor's Letter-box have now reached such unwieldy proportions that it has become necessary to establish a hard and fast rule regarding Answers to Correspondents. In future, all questions requiring replies will continue to be answered in this column without any fee. If an answer is required by letter, a fee of half-a-crown must be enclosed with the note containing the enquiry. We are always pleased to help our numerous correspondents to the fullest extent, and we can trust them to sympathise in the overwhelming amount of writing which makes the new rules a necessity. Every communication must be accompanied by the writer's name and address, otherwise it will receive no attention.

*A Good Book on "Children."*

(1) Can you recommend such a book for the benefit of a young mother? —*Mother.*

"The Mother's Help and Guide to the Domestic Management of Children," by Dr. Murray Braidwood, will probably suit your needs. It is published by The Scientific Press, price 2s.

*Midwife.*

(2) What are the duties of a monthly nurse in the house of a private patient?

Read the chapters on "Midwifery Nursing" in Dr. Percy Lewis's "Nursing: Its Theory and Practice." (Scientific Press, 28 & 29, Southampton Street, Strand, London, W.C.) There you will find all your questions answered in full.

*A Short Term of Training.*

(3) The Matron of the North-Eastern Hospital for Children, Shoreditch, would be glad to know if any hospitals in London will take women who have served for three years as probationer in a children's hospital, for six months or one year's training in adult general nursing?

No general hospital in London will take non-paying probationers for a shorter term of training than two years. Women who can afford to pay can enter at many hospitals for periods of three months, the usual payment being one guinea a week, but of course no certificate of any value would be given for a short time.

*Advice Wanted.*

(4) Please tell me the price of "How to Become a Nurse," and kindly recommend other books for home study. I have passed two of the St. John Ambulance exams., but am told they will not help me at all as a nurse. Would the training at a fever hospital be sufficient to qualify one for entering a general hospital as a nurse?

"How to Become a Nurse" is published at 2s. 6d. Write to the Scientific Press, 28 & 29, Southampton Street, Strand, London, W.C., for a catalogue of books for nurses. You will also find a list at the end of "How to Become a Nurse." For general study get Dr. Percy Lewis's "Nursing: Its Theory and Practice," from the above address. Certainly your studies for the St. John Ambulance exams. will help you in the theory part of your work as a nurse. It is better to go in for general training first and afterwards gain experience in fever nursing. The training at a fever hospital would not qualify you for entering a general hospital in any other capacity but that of a probationer.

*Home for Incurables.*

(5) Can you tell me of a home or hospital for incurables where a man suffering from paralysis could be taken in? I think he would be able to pay a little.—*Sister Annie.*

In Burdett's "Hospitals and Charities" (Scientific Press, 28 & 29, Southampton Street, Strand, London, W.C.) you will find lists of such homes as you require with all particulars as to terms of admission and payment.

*Training.*

(6) Can you advise me how to set about placing two young women, 26 and 30 years of age, in a London hospital as probationers?—*W. W.*

Write to the matrons of general hospitals in London for particulars and forms of application. Just now the staff is being increased at the London hospital, Whitechapel, and the matron will be glad to hear of suitable candidates. You will find lists of the hospitals in Burdett's "Hospitals and Charities."

*Massage.*

(7) Where can a nurse learn massage and gain a certificate in London? Is it an advantage for a nurse with general training to learn massage?—*C. T. K. and S. E.*

Write to the hon. secretary, Society of Trained Masseuses, Trained Nurses' Club, 12, Buckingham Street, Strand. Yes; a knowledge of massage will probably be very useful to you.

*A Book on Nursing.*

(8) I wish to make a nurse a present of a good book on nursing. Please recommend one.

Write to the Scientific Press, 28 & 29, Southampton Street, Strand London, W.C., for their catalogue of nursing books, and choose from this list what will best suit your friend. "Nursing," by Mabel Hampton, price 7s. 6d., is an excellent book.

*Cambridge.*

(9) Please tell me if you know of any nurses' co-operation in or near Cambridge?—*C. S. D.*

We do not know of any.

*Cleansing Powder.*

(10) Can you tell me where Smith's Cleansing Powder can be obtained? —*Cleanliness.*

We should think any chemist could tell you.

## Figures and Tables

**Fig 45. f1:**
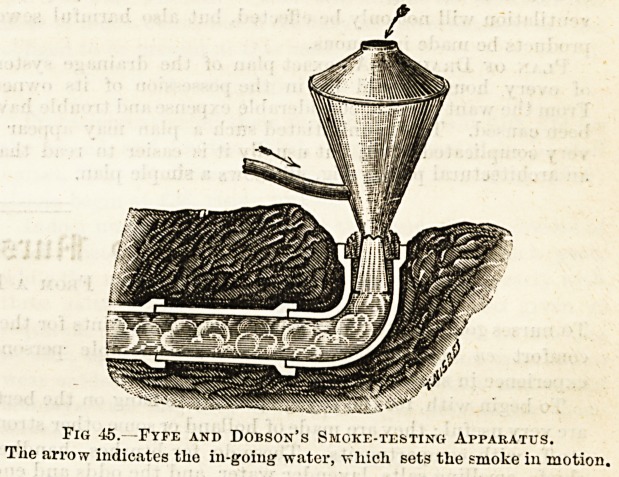


**Fif. 46. f2:**
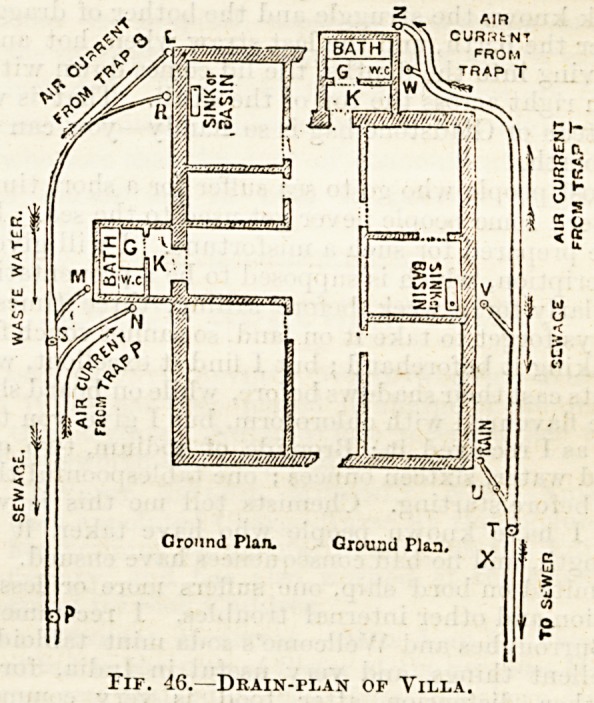


**Figure f3:**